# New Synthetic Compounds with Psychoactive Action—Preliminary Results Among Primary and High School Students on the Territory of Novi Sad

**DOI:** 10.3390/medicines12010006

**Published:** 2025-03-14

**Authors:** Igor Kelečević, Ljubica Gugleta, Ana-Marija Vejnović, Vesna Mijatović Jovin

**Affiliations:** 1 Clinic for Psychiatry, Clinical Center of Vojvodina, 21000 Novi Sad, Serbia; 2 Faculty of Medicine, University of Novi Sad, 21000 Novi Sad, Serbia; 3 Department of Psychiatry and Psychological Medicine, Faculty of Medicine, University of Novi Sad, 21000 Novi Sad, Serbia; 4 Department of Pharmacology, Toxicology and Clinical Pharmacology, Faculty of Medicine, University of Novi Sad, 21000 Novi Sad, Serbia

**Keywords:** novel psychoactive substances, legal highs, drugs, survey, schools, pupils, students, adolescents

## Abstract

**Introduction:** Novel psychoactive substances (NPSs) are substances not controlled by the United Nations’ 1961 Narcotic Drugs and 1971 Psychotropic Substances convention, which pose a threat to public health. The use of NPSs is growing among recreational drug users. NPSs mimic the effects of the existing illegal drugs; they are used as substitutes for the traditional drugs of use. NPSs are commonly marketed as safe substances. NPS abuse is especially risky among vulnerable individuals, such as children and adolescents. **The Aim:** This study aims to analyze the knowledge and attitudes of primary and high school students regarding NPSs, determining the frequency and patterns of NPS use, and examine motivational factors for their consumption. **Methodology:** The questionnaire was employed to primary and secondary school students of the city of Novi Sad in November 2024. The data were analyzed using the methods of descriptive and inferential statistics in the statistical software package JASP 0.18.1.0. **Results:** A total of 1095 participants took part in the survey (53.6% males and 46.4% females). The age range of participants was 11–18 years (mean age 14.637 years). The majority of pupils lived in the city (70.5%). The most numerous students were students with the highest overall grade. The proportion of students who were familiar with NPSs was 38.3%, while 61.7% of them were not aware of their existence. Living in cities correlated positively with the NPS knowledge. The NPS risk awareness was notably low. The proportion of students who tried one or more novel drugs was 1.918%. **Conclusions:** The abuse of novel psychoactive substances is a growing concern, particularly among young individuals, requiring increased awareness and education on their risks. Educational systems should provide accurate information to prevent false beliefs, while policymakers must legally regulate new drugs. A coordinated approach is crucial for effective prevention, involving education, media, and support from different organizations. Future studies should focus on the impact of education on attitudes towards NPSs.

## 1. Introduction

Novel psychoactive substances (NPSs) are defined as new narcotics and/or psychotropic drugs, either in pure form or as preparations, that are not controlled by the United Nations’ 1961 Narcotic Drugs and 1971 Psychotropic Substances conventions, but that may pose a threat to public health, with risks comparable to those of substances listed in these conventions [1,2,3]. The use of NPSs is growing among recreational drug users and there is an ongoing increase in the number of these substances. Once a previous generation of NPSs becomes legally controlled, a new generation usually emerges on the market [4]. Because NPSs have spread to a large number of countries and continents, they are considered a global phenomenon [1,2].

The abuse of NPS has been increasing since the late 2000s [4,5]. These compounds are mostly synthetic and sometimes referred to as “legal highs” because they are usually created in underground laboratories by altering the chemical structure of an existing controlled compound. They are considered legal substances until they are classified as illicit drugs [2,4,5,6]. NPSs are designed to mimic the effects of the existing illegal recreational drugs and they are used as chemical substitutes for traditional psychotropic drugs [4,6]. The users are drawn to them for their psychoactive effects and probably because they cannot be detected in routine screenings [1].

The market for novel psychoactive substances is growing each year [1]. These drugs can be bought over the Internet, in head shops, or from drug dealers. Websites selling these compounds are usually freely accessible, but buying them from the dark web has become popular recently [4]. NPSs are typically sold in packages labeled “not for human consumption” and/or “research chemical” [1]. The development of online drug markets currently exceeds the scientific research on these substances [7]. Most information is currently derived from case reports and case series [6].

Vulnerable individuals (children, adolescents, and psychiatric patients) are nowadays exposed to websites designed for purchasing NPSs and/or providing the information about these drugs [1]. These websites often use aggressive marketing strategies, such as attractive drug names, colorful packaging, and free test samples. They emphasize the psychoactive effects of the substance and assure users that they cannot be detected in routine drug screenings [2]. Drug users can also learn about NPS from drug forums or “e-psychonauts”—individuals who experiment with drugs to induce altered states of consciousness to explore their own mind [1,8,9]. This contributes to a lack of awareness about the risks of NPS consumption, leading many recreational drug users to believe that NPSs are safe because they are legal [2].

The existence of NPSs represents a significant psychiatric issue. The use of these substances affects the action of neurotransmitters and receptors in the central nervous system (CNS), which can lead to psychopathological manifestations [10]. Almost all NPSs can cause serious psychiatric side effects (Table 1), with psychosis being one of the most prominent.

Psychotic disorders among NPS users are not fully understood, and there is ongoing research to better understand these clinical manifestations, particularly given the rising number of studies linking the abuse of NPSs to the onset of psychosis at an earlier age (children and adolescents), as well as more severe courses of illness [11]. Recent studies have largely focused on frequently reported association between synthetic cannabinoids (SCs) and psychosis, whether it involves the exacerbation of existing psychiatric illness or the onset of a first psychotic episode [11,12,13]. The literature includes numerous reports discussing psychotic symptoms following the consumption of SCs, which have stronger effects on the endocannabinoid system in CNS than delta-9-tetrahydrocannabinol (THC), the main psychotropic component of cannabis [11,13]. These findings suggest that SC users may experience more severe psychiatric symptoms (including suicidal ideation) compared to cannabis users, and especially compared to non-users [12]. Genetic predisposition plays a significant role, as some SC users who developed psychosis had a positive family history of mental disorders, such as schizophrenia, mood disorders, and substance use disorders [11]. Some researchers speculate that the abuse of NPSs could lead to treatment-resistant psychosis, requiring clozapine treatment, especially for those individuals who are genetically burdened [11]. Furthermore, the occurrence of acute psychosis following SC use has been studied and termed “Spiceophrenia” by Papanti et al. [5,12,14].

According to the scientific literature, the main groups of NPSs include synthetic cannabinoids, synthetic cathinones, phencyclidine-like dissociative drugs, tryptamines, phenethylamines, piperazines, GABA-A/B receptor agonists, and synthetic opioids. Table 1 provides a summary of these compounds, along with their effects and side effects.

**Synthetic Cannabinoids.** The compounds in this group are variants of THC, the primary cannabinoid found in the plants of the *Cannabis* genus [6,15]. SCs are typically mixtures sprayed onto a dried marijuana-like plant base. Commonly, they are consumed by smoking [1,5,6]. Oral, e-liquid, and injectable formulations have also been described in the literature [1].

SCs are more potent than THC at cannabinoid receptors level in CNS, leading to stronger effects and adverse reactions compared to the natural compound (Table 1) [1,13,14]. Chronic use of these substances can lead to tolerance, dependence, and serious withdrawal symptoms [1]. The abuse of SCs by vulnerable individuals (e.g., adolescents) can trigger de novo psychosis or exacerbate existing psychotic disorder, as mentioned earlier [5,14].

**Synthetic Cathinones.** These substances are chemically and pharmacologically related to cathinone, the active ingredient with psychoactive properties naturally found in the leaves of the khat shrub (lat. *Catha edulis*) [16,17]. They are structurally similar to amphetamine and catecholamines, possessing stimulant, amphetamine-like, properties [1,2]. However, their chemical and pharmacological properties, as well as their potency, differ from amphetamine due to slight variations in their chemical structure [1]. On the market, they are usually sold under false labels like “bath salts” and “plant food”, while some are marketed as legal alternatives to classical recreational drugs (e.g., cocaine and methamphetamine) [16]. Common formulations include powder and pills, and they can be consumed by snorting, oral ingestion, or intravenous injection (sometimes the rectal venous plexus) [1,16]. They gained popularity after legal restrictions reduced the availability of cocaine and MDMA (Ecstasy) [1]. Tolerance, dependence, and withdrawal symptoms can also be seen in users of synthetic cathinones [18].

**Phencyclidine-like Dissociative Drugs.** These drugs belong to a larger group of substances named dissociatives, known for inducing a dissociated state characterized by a feeling of an absence of time, weightlessness, and detachment from one’s own body [6].

They can be administered via injection, snorting, smoking, or rectally [1]. The abuse of these substances can cause serious and long-term unwanted effects (Table 1). Tolerance, dependence, withdrawal signs, and flashbacks have been reported [19].

**Tryptamines.** These drugs are derivatives of the amino acid tryptamine [1]. Tryptamines belong to the class of compounds named psychedelics, known for altering sensory perception and inducing quasi-mystical experiences (Table 1) [6]. Lysergic acid diethylamide (LSD) is a traditional member of this group, and it is considered the most potent known hallucinogen [6,16]. Most of these compounds occur naturally, and they are usually consumed by eating or drinking [1,16,20]. Synthetic derivatives can be taken orally, smoked, sniffed, or injected [21].

**Phenethylamines.** Various NPSs are included in this group, and they are often associated with acute toxicity and fatal outcomes (Table 1) [1].

**Piperazines (“Party Pills”).** Popular among recreational users, these drugs mimic the effects of MDMA (Table 1) [1]. Benzylpiperazine (BZP) is often found in “fake” Ecstasy tablets (“Legal E” and “Herbal Ecstasy” [16]), while recently introduced combination of MDMA and piperazine (“Molly”) mimics the effects of amphetamine (ingestion is associated with stimulant effects and higher doses may induce hallucinations) [1].

**GABA-A/B Receptor Agonists.** These include gamma-hydroxybutyric acid (GHB, “liquid Ecstasy”), gamma-butyrolactone (GBL), and 1,4-butanediol [1]. The abuse of these compounds is relatively low in Europe, but still prevalent among young people [22]. GHB is highly addictive; its withdrawal symptoms can occur rapidly, including insomnia, muscle cramps, tremors, seizures, and psychosis (Table 1) [1,22].

**Opioids.** These novel drugs are synthetic derivatives of classical recreational opioids, mostly fentanyl [4]. Fentanyls are sold as powders, nasal sprays, liquids, or pills [4,6]. New synthetic opioids, though chemically different from fentanyl, have similar mechanisms of action. MT-45 is a popular compound among recreational drug users, often mixed with synthetic cathinones (“Wow”) [4,23,24]. Illicit opioids are frequently mixed with heroin („fake heroin“) or included in cocaine products, and they can even be pressed into fake prescription pills [4]. The desired effects are similar to heroin, but last longer [4,6]. Due to their narrow therapeutic index, synthetic opioids are dangerous. Inexperienced users are at high risk of fatal respiratory depression and pulmonary edema (Table 1) [4].

**Table 1:** List of the groups of Novel Psychoactive Substances, their desired effects, and potential unwanted reactions to their consumption [1,2,4,6,10,14,16,17,20,22,25,26,27,28,29,30,31,32,33].

NPS Group and Important Representatives	Desired Effects	Side Effects
**Synthetic Cannabinoids** *K2* *Krypton* *Aroma* *Bonzai* *Spice* *Noids*	Euphoria, relaxation, stimulation, sedation, anxyolysis, and auditory and visual hallucinations	Psychotic-like symptoms (paranoia, disorganized behavior, delusions, visual and auditory hallucinations, catatonia, depersonalization, and suicidal thoughts), anxiety, agitation, cognitive impairment, encephalopathy, stroke, seizures, midriasis, slurred speech, excessive sweating, hypertension, tachycardia, dyspnoea, nausea, vomiting, heart, lung, and renal failure, coma, accidental deaths, and suicide
**Synthetic Cathinones** *Mephedrone (4-methylmethcathinone, 4-MMC)* *Metaphedrone (3-methylmethcathinone 3-MMC)*	Heightened mood, euphoria, increased alertness and energy, feeling of self-confidence, and elevated sexual drive	Anxiety, agitation, depression, insomnia, hallucinations, paranoid ideations, delirium, hypertension, tachycardia, midriasis, abdominal pain, sweating, chills, hyperthermia, rhabdomyolysis, renal and hepatic failure, and seizures
**Phencyclidine-like Dissociative Drugs** *Ketamine (“Special K”)* *Methoxetamine (“mexxy”)*	Induction of a dissociated state (feeling of an absence of time, weightlessness, and disconnection from one’s own body), “K hole” (near-death experiences) and “M hole” (extreme and long-lasting depersonalization and derealization), and auditory and visual hallucinations	Psychosis, agitation, neurocognitive deficits, cerebellar toxicity, cardiovascular and respiratory toxicity, urological and intestinal problems (“K-bladder” and “K-cramps”), collapse, hypothermia, rhabdomyolysis, and accidental deaths
**Tryptamines** *Lysergic acid diethylamide (LSD)* *Dimethyltryptamine (DMT; 5-MeO-DMT)* *Psilocin/psylocibin (4-OH-DMT)* *Bufotenin*	Psychedelic effects (alteration of sensory perception and quasi-mystical experiences, and depersonalization), stimulation, euphoria, intense feelings, and optical hallucinations	Psychosis, agitation, paranoid ideation, severe mood changes, panic attacks, memory impairment, tachycardia, hypertension, tremor, hyperthermia, rhabdomyolysis, seizures, coma, and death
**Phenethylamines** *Amphetamine* *Metamphetamine* *Paramethoxymethamphetamine* *Methylenedioxymethamphetamine (MDMA)* *2C substances* *B-Fly* *25C-NBOMe* *6-APB*	Stimulant, entactogenic (increasing the feelings of empathy, emotional openness, and connection with other people), dissociation, and hallucinogenic effects	Dysphoria, hallucinations, depression, cognitive impairment, midriasis, tachycardia, hypertension, vomiting, diarrhea, metabolic acidosis, convulsions, hyponatremia, thrombocytopenia, hyperthermia, rhabdomyolysis, liver and kidney failure, and death
**Piperazines** *Benzylpiperazine (BZP)*	Stimulation, euphoria, and hallucinations	Seizures, hyponatremia, hyperthermia, rhabdomyolysis, renal failure, and death
**GABA-A/B Receptor Agonists** *Clonazolam* *Etizolam* *Flubromazepam* *gamma-hydroxybutyric acid (GHB)* *gamma-butyrolactone (GBL)* *1,4-butanediol*	Euphoria, calmness, sedation, anxyolysis, hypotonia (depending on the intensity), lowering of inhibitions, and increased libido	Amnesia, confusion, disorientation, delirium, hallucinations, hypotonia, dizziness, nausea, vomiting, muscle stiffness, slurred speech, blurred vision, convulsions, depression of cardiopulmonary function, and death; withdrawal can include insomnia, muscle cramps, tremor, anxiety, and seizures
**Opioids** *Acetylfentanyl* *Ocfentanil* *Carfentanil* *AH-7921 (“doxylam”)* *MT-45* *U-47700*	Pleasure, enjoyment, euphoria, relaxation, sedation, dissociating effects, and dream-like state	Confusion, disorientation, intense sedation, constipation, nausea, miosis, slurred speech, respiratory depression, pulmonary edema, coma, and fatal outcome

### The Aim

The aim of this study is to analyze the knowledge and attitudes of primary and secondary school students regarding novel psychoactive substances, and to examine these results in relation to demographic factors. This study also seeks to determine the prevalence of NPS use, as well as to assess usage patterns and motivational factors for their consumption.

## 2. Methodology

This research was conducted by employing a questionnaire to pupils in higher grades of primary schools and high school students in the city of Novi Sad. Novi Sad is the second largest city in the Republic of Serbia and the capital of the Autonomous Province of Vojvodina, with a population of 368.967 inhabitants, according to the 2022 census [34]. The questionnaire was administered in November 2024, after obtaining consent from the school principals and agreeing on the organizational procedure. The participants were informed that the survey was anonymous and that no personal information would be collected.

The questionnaire included the necessary information about the main goals of the study, described in an understandable manner, ensuring pupils were adequately informed about the topic. It included questions on socio-demographics (age, sex, and residential setting) and the academic performance (overall grade). The second section focused on knowledge of novel psychoactive substances and eventual consumption of these drugs, including patterns of use.

After the survey was completed in all the consenting schools, the results were analyzed and summarized using descriptive and inferential statistics in the *JASP 0.18.1.0* statistical software. Most variables in the questionnaire were categorical (the grading system, while ordinal, was also considered categorical), and age was the only numerical variable (treated as ordinal). Categorical variables were presented as frequencies, while age was represented using range and mean value. The statistical correlation between variables was calculated using the chi-squared test for categorical variables. The significance level was set at *p* < 0.05. The strength of association was measured using the phi coefficient (for 2 × 2 tables), Cramer’s V (for tables larger than 2 × 2), and Kendall’s tau-b (for ordinal variables).

The binary logistic regression model was considered to determine if a set of predictors (i.e., survey responses) might effectively anticipate a binary outcome (whether or not NPS were used). However, due to a large imbalance between the two outcomes, the model was only able to predict the dominant class effectively, while predictive performance for the smaller class was rather low. Therefore, this statistical method was omitted from the study. Alternative statistical approaches to balance classes might be resampling techniques (oversampling the smaller class or undersampling the dominant class). These methods were considered but excluded due to software limitations.

The study’s methodological correctness according to the principles of the Declaration of Helsinki was confirmed by the Ethical Committee of The Academy for Human Development in Belgrade (Date 12 November 2024; No. 7/363).

## 3. Results

A total of 1095 participants filled out the survey. As no missing data were detected, all of them were included in the study. The participants were evenly distributed by gender, with 587 (53.6%) males, and 508 (46.4%) females. The age range of participants was 11 to 18 years (mean = 14.637; SD = 1.315; 95% CI = 14.559–14.715). The majority of pupils were either 14 or 15 years old (63.0%). A statistically significant, but weak, correlation (*p* < 0.001, Kendall’s Tau = −0.110) was observed between gender and age; the females were slightly older (mean = 14.846; SD = 1.458; 95% CI = 14.719–14.974) than the males (mean = 14.457; SD = 1.149; 95 CI = 14.363–14.550) in our sample. The distribution of participants by age is shown in Table 2.

The majority of pupils in the study live in the city, 772 in total (70.5%), while 323 (29.5%) reside in rural areas.

**Figure 1:**
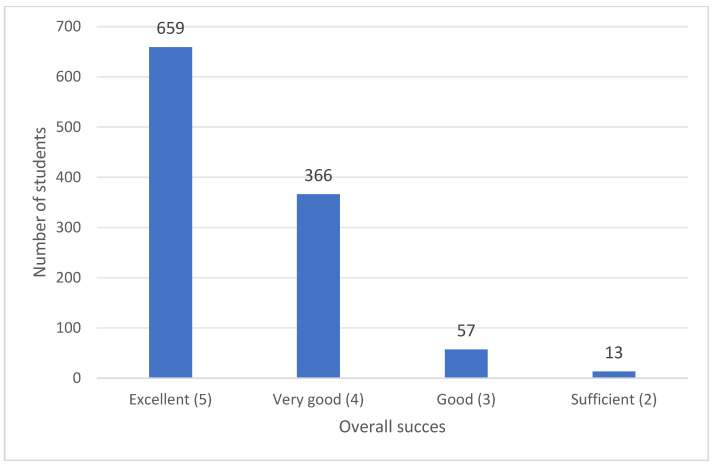
The overall grades of the survey participants.

Among the students in our sample, the highest number were excellent students (having the highest overall grades). None of the students reported an insufficient grade (1). The distribution of pupils according to overall academic performance is shown in Figure 1. The student’s overall success did not significantly correlate with their knowledge of classical drugs or NPSs. However, a considerably high number of excellent and very good students considered NPS abuse to be highly risky, although no statistically significant difference was observed regarding the specific knowledge about the risks that NPSs pose.

A total of 832 students (76%) were familiar with classical drugs, while 263 (24%) had never heard of these substances at the time of the survey. On the contrary, 419 (38.3%) students were aware of NPSs, while 676 (61.7%) were unfamiliar with the term. The students’ answers, categorized by residential area and gender, as well as the statistical test results and effect sizes, are shown in Table 3.

The proportion of students who had tried one or more novel drugs was 21 (1.918%). Of that number, 10 (47.62%) were male and 11 (52.38%) were female, while 16 (76.19%) lived in the city and 5 (23.81%) were from rural areas. The place of residence (urban or rural) was not a significant predictor of NPS use (*p* = 0.564, phi coefficient = 0.017). Additionally, no correlation was found between age and NPS use (*p* = 0.952, Kendall’s Tau = 0.021). However, the majority of students who reported using NPSs were either 14 (N = 6) or 15 (N = 6) years old at the time of the survey, accounting for about 51.14% of this group. These age groups were also the most frequent in our sample (Table 2). A similar observation was made when exploring the distribution of NPS users in relation to academic performance (*p* = 0.337, Kendall’s Tau = 0.030). Among the students with the highest grade (5), 9 (42.86%) reported consuming NPSs, while 10 students (47.62%) with a very good grade (4) also reported using novel drugs. Similar to the age dimension, these grades were predominant in the sample, which explains the results. No students with a sufficient grade (2) reported using NPSs, while two participants (9.52%) with a middle grade (3) answered positively. The patterns of use and motivations among NPS users are shown in Table 4.

A statistically significant correlation was observed in a logical sequence of responses from the survey. First, knowledge about classical recreational drugs correlated positively (*p* < 0.001, phi coefficient = 0.183) with knowledge about NPSs. Second, NPS knowledge was positively correlated with knowing someone who uses NPSs (*p* < 0.001, phi coefficient = 0.222). Finally, knowing an NPS user was a positive predictor (*p* < 0.001, phi coefficient = 0.207) of NPS use.

## 4. Discussion

This research is the first in Novi Sad, Serbia, to analyze the knowledge and attitudes of primary and high school students about novel psychoactive substances. To the best of our knowledge, no similar studies have been performed in the Autonomous Province of Vojvodina so far.

The abuse of NPSs among younger generations is increasing and becoming a widespread phenomenon. Almost 70% of surveyed students agree that the abuse of NPSs is on the increase in Serbia. Not much research on the knowledge and consumption of NPSs in this age group has been done, and results vary among countries [2].

In our sample, around 70% of the participants live in the city, while around 30% live in rural areas. This geographical distribution is similar in proportion to Martinotti et al.’s study, where two-thirds of participants lived in urban areas and one third lived in the countryside [2]. More than 80% of the study sample from the research about the awareness and attitudes of future health care professionals in Serbia, conducted by Mijatović et al., reported living in the city, but their sample included college students, in comparison to our study where younger individuals were included and surveyed [35]. These findings confirm the tested individuals are predominantly urban residents.

When it comes to having knowledge about NPSs or not, we showed that a significant difference exists between urban and rural residents, as it can be expected from city inhabitants to possess knowledge about NPSs, in comparison to people living in smaller areas (e.g., villages) whom are expected not to be as aware about the existence of novel drugs as city inhabitants are. In our study, 38.3% of the pupils reported having previously heard about NPS. Martinotti et al., who investigated the use of NPSs and knowledge among adolescents and young adults in urban and rural areas, found that 53.3% of their sample had previous knowledge about NPSs [2]. This group of authors discussed that the knowledge about NPSs is significantly higher in urban areas, mostly due to peer-to-peer information sharing and the possibility to visit night clubs more frequently in comparison to young people living in the countryside. We could contribute to this discussion by suggesting that NPS awareness and associated consumption risks among young people can spontaneously increase when substance users live in the local area. Young people’s awareness of NPSs in the UK is high (more than 85% in 2017), mainly because of new UK legislation [36]. It can be observed from our study that 33.1% of students who reported living in rural areas possess knowledge about NPSs, which accounts for 9.77% of the entire sample. The other 28.53% of aware students live in the city, which supports the abovementioned presumption about insufficient knowledge in children living in smaller areas. Limited nightlife and social opportunities may reduce the awareness and the risk of consuming these substances. However, the lack of adequate knowledge and sufficient experience, which is important regardless of the residence (urban or rural), and local peer pressures, can lead young people to attempt to consume one of these substances which, in turn, can have devastating effects for the members of this vulnerable population, ranging from mild intoxications, dependence, tolerance, withdrawal syndrome, negative health, family and social outcomes, to severe poisonings with potential disabilities or fatal outcome. Youngsters represent a vulnerable population not only because of their low levels of knowledge, experience and risk awareness, but also due to the fact that their central nervous system has not reached maturity yet; it is speculated that the abuse of NPSs can interact with the CNS maturation processes and lead to permanent alterations of its structure and function, making the young drug users prone to developing different mental health problems (e.g., psychosis, as previously mentioned) or the exacerbation of previously existing psychiatric disorders [37,38].

All of the abovementioned represent serious arguments for implementing continuous educational activities in and out of schools, as well as improving preventive measures and creating new ones in all segments of society.

We hold that the low NPS awareness among the schoolchildren of Novi Sad is due to several factors. While we believe that education programs in our schools include content focused on classical psychoactive substances, it is, on the other hand, presumed that the insufficient quantity of lectures concerning novel drugs is one of the reasons for the awareness discrepancy between classical and novel substances (75.98% vs. 38.3%). Another factor might be insufficient funding for NPS and health education programs in schools and other institutions. Deligianni et al. mentioned media as the most important source of information according to non-users [36]. TV and Internet can provide information to the public regarding side effects, health risks, and dangers associated with the abuse of NPSs. The insufficient (and inappropriate) media attention [38] and lack of public discussion about novel drugs, partly due to social stigma in our area, is concerning. Due to these shortcomings, young people, especially those without strong family and social support, are more likely to obtain information about these substances from inexperienced users and unverified sources of knowledge which are often misleading and inaccurate [36,37].

In our study sample, almost 2% of the participants reported experimenting with NPSs. In agreement, the latest data from the European School Survey Project on Alcohol and Other Drugs Report (ESPAD Report 2019) showed that 3.4% (from 0.9% in Finland and Portugal to 6.6% in Estonia) of schoolchildren used novel drugs, while this percentage in Serbia was 1.8% [3]. Biliński et al. conducted an epidemiological study in order to determine the scale of abuse of designer drugs amongst Polish youth. In their study, 4.49% of school pupils admitted using designer drugs which is a slightly bigger percentage in comparison to our study [39]. Martinotti et al. conducted research among young people in Italy. They registered a global NPS use of 4.7% in 2015, whereas, in 2014, this percentage was 3%, implying a slight increase in users of these drugs [2,40]. Unlike the age range of our study, they included individuals aged from 16 to 24 years, which might interfere with the comparison of results they obtained [2]. Even though the sample size for minors in a UK study by Deligianni et al. was small (13 survey participants), a significant proportion of underage individuals (N = 8) reported having used novel drugs [36]. These percentage differences across studies can be due to the availability of drugs in different regions, the uneven age groups in these studies, and various sample sizes.

Considering the number of participants who know about NPSs and those who tried these drugs, it seems that novel psychoactive substances are gaining popularity among young people.

To support this assumption, Mijatović et al. conducted a study in 2017, in the same region as our research, using a sample of medical students. Their results showed that 23.47% of the participants reported knowing about NPSs [35]. This can be compared to the fact that almost 40% of the pupils in our study heard about these compounds. Additionally, at the time Mijatović et al. conducted their study, only two students (0.41%) reported using NPSs in their lifetime [35].

When asked about motivational factors, “other reasons” was the most common answer, followed by the feeling after consumption and social pressure (“my friends used them”). A couple of authors from Sweden found that the primary motivation for using NPS was exactly for pleasure and enjoyment, similar to our findings [41]. Several other studies confirm the notion that “enjoying intoxication” and “having a good time” are key motivators to NPS abuse [42,43]. The other important aspects of motivation, according to Sande, such as legality and ease of access, were not as significant to the participants in our sample of pupils who reported using NPSs [44]. Similar motivators are discussed in a paper by Deligianni et. al. This group of authors from United Kingdom evaluated patterns of NPS use in a self-selected sample of UK consumers, where the majority of participants were young people (having less than 25 years). In their paper, the main motivator for NPS use was to get high (76.1%). Friends taking them, ease of acquisition, and not being against the law were present at much higher percentages than our study (68.7%, 43%, and 26.9%, respectively) [36].

Our study showed that the majority of users obtained novel drugs from a friend or by purchasing them from a dealer. The previously mentioned couple of authors from Sweden conducted a study to explore the characteristics, attitudes, and motivations, of a self-selected sample of international NPS users [41]. The most common way of obtaining NPSs, in their investigation, was the Internet (60.4%), while friends (17.8%) and dealers (9.5%) were in the second and third place. According to a study conducted by Sande, in Slovenia, the majority of participants also obtained novel drugs from friends and dealers [44]. It seems that recreational drug users start to rely on dealers after the drug becomes illegal [45]. Our results are also in accordance with a group of authors from the UK, in whose sample, the three most common sources of NPSs were drug dealers, the Internet, and friends, respectively [36].

The group of participants who reported using NPSs stated that they usually consumed them with friends and/or when going out. Similarly, in the aforementioned study from the UK, the most common settings of NPS use were at home with friends, house parties, and clubs/raves, while consuming NPSs at home alone was not as frequent, like in our study [36].

The participants in the sample from research by Mijatović et al. were asked if they believed that the psychoactive action of NPSs is unpredictable and can even cause death; about 47% answered positively, and about 52% of them answered that they do not know, while only 10 students (2.04%) disagreed [35]. The highest number of subjects in our study shared similar attitudes when asked about the risk of consumption of NPSs. A study sample from the UK, including mostly younger generations, showed that the perception of high risk from NPS use decreased from 2015 to 2017 (from 35% to 17%) [36]. The lack of risk awareness was seen in every age group in this study. However, the most frequent opinion of individuals of our study is that cases of NPS poisoning are not rare.

While a large number of primary and high school pupils in our cohort consider the use of NPSs to be highly risky, not many of them are familiar with the risks related to the abuse of novel drugs and how to protect themselves from these risks (61% and 80%, respectively), even though the majority of students have excellent or very good overall success in school. This phenomenon was observed among NPS drug users in a study by Deligianni et al. who noticed a significant knowledge gap on these health risks. This was partly explained by the following assumption: the fact that NPSs are highly risky drugs may seem an attractive feature, especially among younger adolescent users, who are willing to take the risks in order to gain new experience from NPSs [36]. Admittedly, it could be argued that risk-awareness exists to some extent, as 48.49% of the participants believe that NPS use is equally or more dangerous than the use of classical drugs. However, 48,04% of the participants were unaware of such differences between these two types of substances, so it is clear that the level of awareness and knowledge about NPSs remains low among young people. It could be expected that, as adolescents get older, a bigger proportion of individuals will believe that NPSs are not safer than classical recreational drugs, as deducted from the findings by Corazza et al. [42].

Having considered the abovementioned facts, the potential prevention strategies should be aimed at reducing the spread of wrong information and sharing the correct and reliable knowledge about these substances [2], whether over the TV and Internet, or by directly giving presentations at schools in order for students to be correctly informed about NPSs and their harmful aspects.

There are several limitations to this study which are considered important. Firstly, this research focused solely on the patterns of abuse of novel psychoactive substances, while attention was not given to potential polydrug abuse in the population of primary and high school students. The abuse of multiple substances and/or smoking may influence the results, and it could be positively correlated with the consumption of NPSs [2]. Secondly, the questionnaire in this study was not formally validated, and no pilot studies were conducted prior to our investigation. We acknowledge that this shortcoming may have affected the validity and reliability of the results, so we suggest that readers interpret them with caution. Thirdly, since the obtained data for our study were collected using the survey filled out by primary and secondary school students, giving socially desirable answers and inaccurately recalling the provided information cannot be excluded from the discussion. Finally, the distributed questionnaire did not include questions about socioeconomic status, family setting, and potential mental health conditions of the participants. If the aforementioned data were included in the questionnaire and the consequent analysis, there is a possibility that this might have influenced the obtained results. We strongly suggest including these questions in further studies.

## 5. Conclusions

The abuse of novel psychoactive substances (NPSs) is a growing public health concern, especially among younger generations. To address this issue, raising awareness of the risks NPSs pose is essential for effective prevention. Education in primary and secondary schools should provide accurate information to prevent the development of misconceptions regarding these substances. Policymakers must stay up-to-date with emerging psychoactive substances and ensure the legal control of novel drugs to reduce their accessibility. Society plays an important role in shaping young people’s attitudes through public discussions and media (e.g., TV and the Internet), where careful reporting is necessary to avoid unintended negative effects [46]. Educational institutions cannot act alone; they require adequate funding, governmental support, and collaboration with non-governmental organizations (NGOs), mental health organizations, and continuous communication with parents and children. We believe there is a need for a coordinated approach focusing on public awareness and educational programs to reduce the abuse of NPSs and associated risks. Future studies should examine the effectiveness of educational programs in shaping students’ attitudes toward NPSs and their impact on prevention.

## Figures and Tables

**Table 2:** The age distribution of the survey participants.

Age (Years)	Number (N)	Frequency (%)	Cumulative (%)
11	1	0.091	0.091
12	42	3.836	3.927
13	154	14.064	17.991
14	314	28.676	46.667
15	376	34.338	81.005
16	93	8.493	89.498
17	83	7.580	97.078
18	32	2.922	100.000
Total	1095	100.0	

**Table 3:** Survey responses from students categorized by gender and residential area.

Question	Answer	Gender		Sig. (*p* =)	Effect Size (Phi/Cramer’s V/Kendall’s Tau-b)	Residential Area		Sig. (*p* =)	Effect Size (Phi/Cramer’s V/Kendall’s Tau-b)
		Male (N, %)	Female (N, %)			Urban (N, %)	Countryside (N, %)		
Are you familiar with classical recreational drugs?	Yes	433 (73.8)	399 (78.5)	0.076	0.056	596 (77.2)	236 (73.1)	0.166	0.044
	No	154 (26.2)	109 (21.5)			176 (22.8)	87 (26.9)		
Are you familiar with novel psychoactive substances?	Yes	222 (37.8)	197 (38.8)	0.792	0.010	312 (40.4)	107 (33.1)	0.028	0.068
	No	365 (62.1)	311 (67.2)			460 (59.6)	216 (66.9)		
Do you consider the abuse of NPSs to be on the increase in Serbia?	Yes	392 (66.8)	356 (70.1)	0.269	0.035	505 (65.4)	243 (75.2)	0.002	−0.096
	No	195 (33.2)	152 (29.9)			267 (34.6)	80 (24.8)		
Do you know where the NPSs are mostly used?	Yes	149 (25.4)	124 (24.4)	0.763	−0.011	198 (25.6)	75 (23.2)	0.441	0.026
	No	438 (74.6)	384 (75.6)			574 (74.4)	248 (76.8)		
Do you know the reasons why NPSs are consumed?	Yes	145 (24.7)	178 (35.0)	<0.001	0.113	246 (31.9)	77 (23.8)	0.010	0.080
	No	442 (75.3)	330 (65.0)			526 (68.1)	246 (76.2)		
Does anyone you know use NPSs?	Yes	95 (16.2)	109 (21.5)	0.031	0.068	148 (19.2)	56 (17.3)	0.532	0.021
	No	492 (83.8)	399 (78.5)			624 (80.8)	267 (82.7)		
Do you know how NPSs are consumed?	Yes	102 (17.4)	81 (15.9)	0.581	−0.019	139 (18.0)	44 (13.6)	0.092	0.054
	No	485 (82.6)	427 (84.0)			633 (82.0)	279 (86.4)		
Are you familiar with the risks the abuse of NPSs carries?	Yes	208 (35.4)	219 (43.1)	0.011	0.078	315 (40.8)	112 (34.7)	0.068	0.057
	No	379 (64.6)	289 (56.9)			457 (59.2)	211 (65.3)		
In comparison to classical drugs, I think that the consumption of NPSs is…	Safer	23 (3.9)	15 (3.0)	0.303	0.058	25 (3.2)	13 (4.0)	0.233	0.063
	Equally dangerous	202 (34.4)	193 (38.0)			293 (38.0)	102 (31.6)		
	More dangerous	81 (13.8)	55 (10.8)			95 (12.3)	41 (12.7)		
	I do not know	281 (47.9)	245 (48.2)			359 (46.5)	167 (51.7)		
I think that the risk of consumption of NPSs is…	Low	24 (4.1)	14 (2.8)	0.505	0.046	23 (3.0)	15 (4.6)	0.210	0.064
	Moderate	72 (12.3)	64 (12.6)			104 (13.5)	32 (9.9)		
	High	268 (45.6)	248 (48.8)			365 (47.3)	151 (46.8)		
	I do not know	223 (38.0)	182 (35.8)			280 (36.2)	125 (38.7)		
Are you familiar with ways of protecting yourself from the risks that NPSs carry?	Yes	117 (20.0)	101 (19.9)	1.000	−6.240 × 10^−4^	150 (19.4)	68 (21.1)	0.596	−0.019
	No	470 (80.0)	407 (80.1)			622 (80.6)	255 (78.947)		
Are the cases of poisoning by NPSs rare?	Yes	164 (28.0)	115 (22.6)	0.133	0.061	183 (23.7)	96 (29.7)	0.079	0.068
	No	350 (59.6)	325 (64.0)			483 (62.6)	192 (59.5)		
	I do not know	73 (12.4)	68 (13.4)			106 (13.7)	35 (10.8)		

**Table 4:** The users’ reported patterns of consumption and motivational factors.

Question	Answers	N (%)
Frequency of NPS use	Once	10 (47.6)
	Tried NPSs several times	7 (33.4)
	Once weekly or more often	2 (9.5)
	Prefer not to answer	2 (9.5)
Reason to use NPSs	They are safer than classical drugs	1 (4.76)
	I was curious	1 (4.76)
	I liked what I felt after the consumption	5 (23.80)
	They help me socialize/connect with others	1 (4.76)
	My friends used them	5 (23.80)
	They were easy to obtain	1 (4.76)
	I know I did nothing illegal	1 (4.76)
	Other reasons	7 (33.4)
	Prefer not to answer	2 (9.52)
In which situations did you use NPSs?	With friends	10 (47.62)
	When alone	2 (9.52)
	When going out	5 (23.80)
	Other	6 (28.57)
	Prefer not to answer	2 (9.52)
How did you obtain NPSs?	From a friend	10 (47.62)
	From boyfriend/girlfriend	1 (4.76)
	Purchased from a dealer	4 (19.05)
	Purchased from a website	2 (9.52)
	Other	4 (19.05)
	Prefer not to answer	2 (9.52)

## Data Availability

The raw data supporting the conclusions of this article will be made available by the authors on request.
